# A new ecotoxicological test method for genetically modified plants and other stressors in soil with the black fungus gnat *Bradysia impatiens* (Diptera): current status of test development and dietary effects of azadirachtin on larval development and emergence rate

**DOI:** 10.1186/s12302-018-0167-8

**Published:** 2018-09-27

**Authors:** Stephan Jänsch, Johannes Bauer, David Leube, Mathias Otto, Jörg Römbke, Hanka Teichmann, Karolina Waszak

**Affiliations:** 1 ECT Oekotoxikologie GmbH, Böttgerstr. 2-14, 65439 Flörsheim, Germany; 2 Federal Agency for Nature Conservation (BfN), Konstantinstr. 110, 53179 Bonn, Germany

**Keywords:** Risk assessment, Genetically modified organism (GMO), Systemic pesticide, Terrestrial invertebrate toxicology, Veterinary pharmaceutical, Test development

## Abstract

**Background:**

Few suitable and standardized test methods are currently available to test the effects of genetically modified plants (GMP) on non-target organisms. To fill this gap and improve ecotoxicological testing for GMP, we developed a new soil ecotoxicological test method using sciarid larvae as test organisms.

**Results:**

*Bradysia impatiens* was identified as a candidate species. Species of the genus *Bradysia* occur in high numbers in European agroecosystems and *B. impatiens* can be reared in the laboratory in continuous culture. A functional basic test design was successfully developed. Newly hatched larvae were used as the initial life stage to cover most of the life cycle of the species during the test. Azadirachtin was identified as a suitable reference substance. In several tests, the effects of this substance on development time and emergence rate varied for different temperatures and test substrates. The toxicity was higher at 25 °C compared to 20 °C and in tropical artificial soil compared to coconut fiber substrate.

**Conclusions and outlook:**

Results suggest that the developed test system is suitable to enter a full standardization process, e.g., via the Organisation for Economic Co-operation and Development. Such a standardization would not only assist the risk assessment of GMP, but could include other stressors such as systemic pesticides or veterinary pharmaceuticals reaching the soil, e.g., via spreading manure. The use of sciarid flies as test organisms supports recommendations of EFSA, which stressed the ecological role of flies and encouraged including Diptera into test batteries.

## Background

With modern biotechnology, crop plants with new combinations of genes and novel attributes are created that would otherwise not occur under natural conditions. Hence, adverse effects of such genetically modified plants (GMP) on human beings and the environment must be considered [10]. Artificially introduced genes and their products and possible unintentional changes to the metabolic pathways of GMP may produce substances or combinations of substances that are harmful to non-target organisms (NTO). Before a GMP may be released into the environment and placed on the market in the European Union (EU), an environmental risk assessment (ERA) is required [10, 11]. State-of-the-art ecotoxicological test methods investigating the adverse effects of GMP on the environment should be designed for the specific characteristics of GMP and are an important component of the ERA [12, 13]. However, even 25 years after the first registration proposals for GMP were submitted, a standardized test method specifically developed for this purpose remains unavailable.

Currently, testing of the effects of GMP on NTO is primarily based on various ecotoxicological test methods originally developed and standardized for assessing the effects of plant protection products (PPPs) [43]. However, most of the hitherto existing ecotoxicological test methods do not meet the specific characteristics of the assessment of GMP, including the exposure of NTO via food and the food chain. Furthermore, there are limited possibilities to perform dose–response tests with GMP material and to apply safety factors on the derived effect concentrations. Additionally, Directive 2001/18/EC [10] explicitly requires a ‘case-specific’ ERA. Annex II of the Directive defines a case as a combination of the parent organism (the crop plant and its biology, ecology and agronomy), its genetic modification and the possible receiving environment (i.e., a combination of abiotic factors and the biocoenosis) related to the intended (as opposed to accidental) release and use of the GMP. These registration requirements for GMP significantly exceed those for PPPs. A critical issue when accounting for the receiving environment is the choice of test organisms. Hilbeck et al. [20] pointed out that the use of universal standard test organisms for the assessment of chemicals may not meet the case-specific requirements for the assessment of GMP. Test species for PPP often do not occur in the receiving environment. Together with the need to consider exposure via food containing or consisting of GMP, there is a need for additional test methods that account for these differences. Newly selected species must be practical for use in standardized testing under laboratory conditions, e.g., laboratory breeding must be possible [42].

In this context, we present a new test system specifically designed for the assessment of GMP effects on non-target soil organisms. While the first part of this publication deals with the species selection and the development of the test system, the second part reports and discusses the test performance and results with the reference substance azadirachtin. Finally, conclusions are drawn and an outlook on the next steps in the further development and standardization process is given.

## Selection and description of the test species

### Selection process

Hilbeck et al. [21] described a structured, stepwise selection procedure for the identification of organism groups potentially suitable as test species for the assessment of GMP. Applying this procedure for the receiving environments of Germany, larvae of the dipteran family Sciaridae were identified as a potential test taxon because of their ecological relevance in Central European agricultural soils [8, 33]. Similar to other soil dwelling dipteran larvae, Sciaridae can reach high numbers in species and individuals in agricultural fields in the temperate regions of Central Europe [29] and participate in organic matter decomposition. A comprehensive appraisal of the ecological importance and ecosystem function of soil dwelling Diptera, including Sciaridae, is given by Frouz [16]. Of the worldwide 1700 species [30], approximately 340 species occur in Germany [29]. Whereas *Lycoriella castanescens* (Diptera: Sciaridae) has been listed as a promising candidate species [21], no culture of *L. castanescens* was available at the start of the study. However, for another sciarid species, *Bradysia impatiens*, a culture existed at the Julius-Kühn-Institute (JKI) in Kleinmachnow (Germany). Büchs [6, 7] performed tests with *B. impatiens* and *Bacillus thuringiensis* (*Bt*) maize and confirmed the exposure of the larval stage to the *Bt* toxin from MON810 maize. *Bradysia* spp. are found in agricultural fields at abundances similar to those of *L. castanescens* [8, 33], but no published records of the species *B. impatiens* from agricultural fields exist [19]. For the test development, *B. impatiens* was used as there is no reason to believe that both sciarid species differ in terms of susceptibility to GMP material. Both are destruents of soil organic matter, feed on fungi and have similar life history parameters [4, 9, 24, 29].

### Biology of *Bradysia impatiens*

*Bradysia impatiens* (Johannsen, 1912) (= *B. difformis* Frey, 1948 = *B. paupera* Tuomikoski, 1960 = *B. agrestis* Sasakawa, 1978) imagines range in size from 1 to 3 mm and are black with long, beaded antennae [26]. *B. impatiens* can be identified based on the characteristic features of the wings, palpi, coxen (tibia) and genitalia [31]. Adults feed only on liquid food [29], whereas larvae are primarily mycophagous but will feed on plant matter when necessary [24]. The gnats prefer phytopathogenic fungi such as *Botrytis cinerea*, *Fusarium* spp. and *Phoma betae* or compost fungi such as *Alternaria alternata* and *Fusarium filiferum* as food sources and for oviposition [26]. Imagines of *B. impatiens* reach sexual maturity shortly after hatching and only live for 2–10 days, depending on the temperature [26, 29]. In a greenhouse, i.e., at 20 °C and 70% relative humidity, Berg [4] determined a survival time of 2–7 days (mean 5.5 days) for imagines. Under such conditions, the duration of development stages was 4–9 days for eggs (mean 6.5 days), 11–17 days for larvae (mean 14 days), and 4–15 days for pupae (mean 4.5 days). Wilkinson and Daughtery [48] found that the highest temperature at which eggs hatch was 32.2 °C and the lowest was between 10 and 12.8 °C. Furthermore, these authors found that the fastest cumulative DT from egg to emergence of adult was 19.2 days, detected under a variable temperature of 18.4–30.0 °C during each 24 h period. The slowest DT was 48.8 days, measured at a constant temperature of 12.8 °C.

### Culturing method

Larvae for starting the laboratory culture at ECT Oekotoxikologie GmbH (ECT) were provided by JKI Kleinmachnow (Germany). The species identity was confirmed morphologically and by barcoding performed by AllGenetics & Biology SL (A Coruña, Spain) [3].

The culturing method used in the present study is based on Kühne and Heller [26]. Coconut fiber was used as substrate (CFS; e.g., Exo Terra^®^ Plantation Soil) and was defaunated by deep-freezing briquettes at temperatures ≤ − 18 °C for at least 24 h. Rearing cages (35 × 35 × 60 cm, Aerarium size M; Papa Papillion) were each equipped with two stainless steel gastronomical containers filled with substrate. For substrate preparation, 200 g of frozen CFS was mixed with 1 l of water, resulting in approximately 2.9 l of moist substrate. Moistened CFS is biologically inert and requires additional organic material to promote fungal growth. The pre-moistened substrate for one container was mixed with 100 g of shredded oatmeal (average grain size 4 mm), resulting in a substrate depth of approximately 5 cm in the containers. After a large number of adults hatched in a cage, the content of one of the CFS containers was replaced. The temperature and humidity in the culturing room ranged from 20 to 30 °C and 50 to 70%, respectively, and the light cycle was set to 12/12 h. Humidity loss through evaporation was compensated for every working day by spraying water on the substrate surface.

## Test development

### Existing approaches from literature and basic considerations

Little previous experience regarding test methods using *B. impatiens* as a test organism was available. Test conditions described in Büchs [6] resulted in pupation rates and emergence rates (ER) of 33% and 12%, respectively, and thus were not suitable to obtain reliable test results. Kühne et al. [27] developed a test method for assessing the level of infestation of *B. impatiens* (as “*B. difformis*”) in growth media for organic farming. This test setup could be adapted to address other questions such as the assessment of the effects of GMP on the reproduction of *B. impatiens*. However, the test method developed by Kühne et al. [27] treats the test substrate as a “black box” that is freely chosen by females for oviposition. Whether any observed effect is a result of choice or avoidance of egg deposition or whether any developmental stage is subsequently affected remain unknown, meaning that standardization and evaluation of the test design from Kühne et al. [27] are problematic, for example regarding the variable number of parent individuals in the rearing cages.

For these reasons, we did not build upon these two test ideas, but decided to use existing accepted standard test methods as a starting point, in particular the chironomid toxicity test according to OECD guideline no. 218 [34] and the dung fly test (OECD guideline no. 228 [37]). In analogy to OECD guideline no. 218, we used 20 synchronized larvae per test vessel, four replicate test vessels, adult emergence and development time as end points, and a validity criterion of 70% emergence in the controls. The type and cover of test vessels was adopted from OECD guideline no. 228. Thus, our tests were performed starting with a previously defined number of larvae to ensure i) the exposure of sensitive live stages and ii) to facilitate a standardized and replicated test performance.

### Test substrates

Two different test substrates were evaluated: coconut fiber substrate (CFS) and tropical artificial soil (TAS).

#### Coconut fiber substrate

CFS consists of compressed coconut husk fiber from plantations in tropical Asia and is commercially available in pet shops. Its physicochemical properties are shown in Table 1 and were determined by Landwirtschaftliche Untersuchungs- und Forschungsanstalt Speyer (Germany). CFS analyses showed low contents of basic nutrients such as phosphorus and ammonium, a very high organic carbon content and a very low nitrogen content. These properties combined with the low palatability of CFS due to its fibrous structure make this substrate an unsuitable food source for *B. impatiens*. Thus, CFS does not compete with an organic (carrier) material to be tested such as oatmeal or GMP material.

**Table 1:** Analysis of coconut fiber substrate used for the rearing and testing of *Bradysia impatiens*

Parameter	Value	Unit
Dry mass	17.1	% FM
pH (CaCl_2_)	5.2	–
Phosphorus	21	mg P_2_O_5_/L FM
Ammonium	0.3	mg/L FM
Nitrate	0.1	mg/L FM
Potassium	612	mg K_2_O/L FM
Magnesium	58	mg Mg/L FM
Salt	1.21	g salt/L FM
Organic carbon	44.5	% C DM
Calcium carbonate	< 0.3	% CaCO_3_ DM
Soluble nitrogen	0.3	mg N/L FM
Bulk density	344	g/L FM

*DM* dry mass, *FM* fresh mass

#### Tropical artificial soil

Artificial soils are widely used in soil ecotoxicological tests such as the OECD collembolan reproduction test [38]. Because in our case the organic material would compete with the plant material to be tested as a food source, the 5% peat foreseen in standard artificial soils was substituted with CFS. To account for this change, the resulting medium was named tropical artificial soil (TAS) in analogy to similarly modified artificial soils [17].

### Fungal inoculum

In early tests during the development process of this test method, the variation in adult emergence rate between replicates of the same treatment was often high (e.g., coefficient of variation ~ 35%). Additionally, characteristics of the fungal growth differed among replicates, both in color and growth habit. To enable a more homogeneous fungal growth in all replicates and thus possibly reduce the variation within treatments, the test substrate was inoculated with the fast-growing mold fungus *Alternaria alternata* (order Pleosporales). It favors decomposing plant material [47] and is very attractive for egg deposition of *B. impatiens* [27].

The *A. alternata* starter culture (provided by JKI Kleinmachnow, Germany) arrived in a Petri dish growing on potato dextrose agar (PDA) and was stored in a thermostatic climate cabinet at 20 °C. Showing the best growth and conidia production by visual examination, Sabouraud agar was chosen for breeding. Three small pieces of mycelium were cut from the starting colony and put in Petri dishes with Sabouraud agar under a fume hood. These were sealed with Parafilm^®^ (Bemis Corporate) and placed in a thermostatic cabinet at 27 °C with an alternate cycle of 12 h light and 12 h darkness [22]. After several days, growth was visible in all Petri dishes.

### Basic test setup

**Fig. 1:**
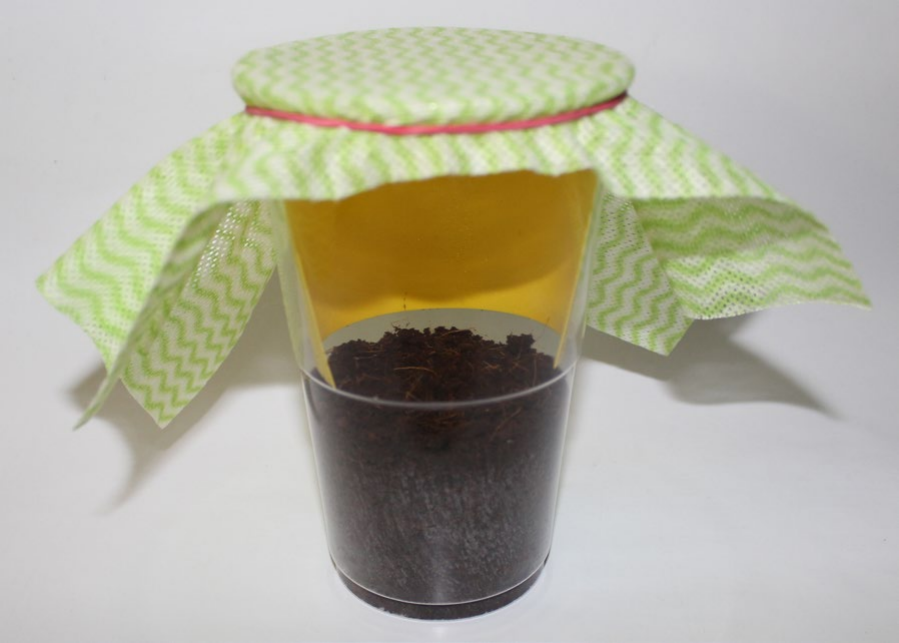
Example of a suitable test vessel covered with cotton cloth and equipped with yellow sticky traps

Test environmental parameters were based on the established culture conditions to provide optimum controlled conditions for the development of the test organisms. Newly hatched larvae were used as the initial life stage, and the adult emergence rate (ER) and development time (DT) were chosen as test end points. To obtain synchronized test organisms from the stock culture for an adult emergence test, Petri dishes containing water agar and oatmeal were placed inside a rearing cage for females to deposit eggs. After 24 h, the dishes were retrieved, closed and stored in a climate cabinet at the same temperature as the subsequent test. At 20 °C, dishes and eggs were stored 9–11 days before the start of the test to allow the larvae to hatch and reach the desired larval stage. Half-liter transparent polyethylene cups (upper diameter: 8.8 cm; lower diameter: 6.0 cm; height 14.5 cm) were used as test vessels. Each test vessel was filled with 100 ± 5 g of moistened CFS and 5 g of oatmeal, acting as a surrogate for what would otherwise be the GMP material to be tested. Test vessels were supplied with 20 larvae, and a 7.5 × 20 cm yellow sticky trap that was placed approximately 1 cm above the substrate surface. Finally, the test vessels were covered with a breathable fabric and closed with a rubber band (Fig. 1). The initial weight of the test vessels was documented, and they were placed inside a climate cabinet at a controlled temperature of 20 °C ± 2 °C or 25 °C ± 2 °C and a light cycle of 12/12 h or 16/8 h light/dark. Twice per week (every 2–4 days), the test vessels were weighed and refilled with distilled water to maintain the initial weight. When emergence was expected, test vessels were checked daily for newly hatched individuals caught on the yellow sticky traps. The test was concluded when no additional adult fungus gnats had emerged for three consecutive days. Test results were considered to be valid when in the untreated control, the mean ER of adults was ≥ 70% of the introduced larvae and began after 12 ± 2 days at 20 °C. A validity criterion for the start of emergence at 25 °C could not yet be assigned and thus requires further study. Substrate samples for determining moisture and pH were collected from each treatment.

### Reference substance azadirachtin

The neem tree, *Azadirachta indica* A. Juss (Meliaceae), originates from southern Asia and today also occurs in parts of Africa, the Americas and Australia [44]. The tetranortriterpenoid compound azadirachtin [25] is presumed to be the most important active component [23].

Insecticides containing azadirachtin operate systemically [25] against numerous economically important insect pests [45]. Azadirachtin is a potent antifeedant and inhibits reproduction by disrupting the synthesis and release of ecdysteroids and other classes of hormones [18, 32] in more than 200 species of insects.

Azadirachtin meets the general requirements for a reference substance [15, 41]. It is specifically recommended for the control of black fungus gnats such as *B. impatiens* by the producer, is plant derived and can be administered through food. Moreover, this compound is readily available commercially and is largely nontoxic to humans [5].

### Test evaluation and data analysis

Emergence rate (ER) and development rate/time (DR/DT) from test start to full adult emergence were assessed as end points. Due to the similarity in test design, the evaluation followed OECD guideline no. 218 [34] as follows:

1 $${\text{ER}} = \frac{{n_{\text{e}} }}{{n_{\text{a}} }},$$

where ER is the emergence rate; $n_{\text{e}}$ is the number of fungus gnats that emerged per vessel; $n_{\text{a}}$ is the number of larvae introduced per vessel.

DR represents the portion of adult development that occurs per day (unit: 1/day) and is the reciprocal of DT, which is the time span between the introduction of eggs (day 0 of the test) and the emergence of the adult flies. The mean DR per vessel ($\overline{x}$) was calculated as follows:

2 $$\overline{x} = \sum\limits_{i = 1}^{m} {\frac{{f_{i} x_{i} }}{{n_{\text{e}} }}} ,$$

where $\overline{x}$ is the mean DR per vessel; *i* is the index of inspection interval; *m* is the maximum number of inspection intervals; $f_{i}$ is the number of fungus gnats emerged in the inspection interval *i*; $n_{\text{e}}$ is the total number of fungus gnats that emerged at the end of the experiment ($= \sum {f_{i} }$); $x_{i}$ is the DR of the fungus gnats that emerged in interval *i.*

3 $$x_{i} = {\raise0.7ex\hbox{$1$} \!\mathord{\left/ {\vphantom {1 {\left( {day_{i} - \frac{{l_{i} }}{2}} \right)}}}\right.\kern-0pt} \!\lower0.7ex\hbox{${\left( {day_{i} - \frac{{l_{i} }}{2}} \right)}$}},$$

where *day*_*i*_ is the inspection day (days since application); *l*_*i*_ is the length of inspection interval *i* (days, usually 1 day).

The no-observed-effect concentration (NOEC) and lowest-observed-effect concentration (LOEC) for ER and DT were determined with the software ToxRat Professional version 2.10.05 (ToxRat Solutions 2010). Control without solvent (C0) and solvent control (SC) data were compared by Fisher’s exact binomial test (two-sided; *α* = 0.05) for ER and for DT by Student *t* test for homogeneous variances (two-sided; *α* = 0.05). For ER (not-emerged individuals), test concentrations were compared to the SC by Fisher’s exact binomial test with Bonferroni correction (one-sided greater; *α* = 0.05) or *χ*^2^ 2 × 2 table test with Bonferroni correction (one-sided greater; *α* = 0.05). For DT, raw data were tested for normal distribution of errors by Shapiro–Wilk’s test (one-sided smaller; *α* = 0.05) and for variance homogeneity by Levene’s test (*α* = 0.05). Comparison of test concentrations with the SC was performed by Williams’ multiple sequential *t* test procedure (one-sided greater; *α* = 0.05).

Concentration–response curves for ER and DT were fitted by three- and four-parameter log-logistic models, respectively, with the free software R version 3.5.1 [39] using the most recent version of the package “drc” [40]. For each concentration–response curve, the upper limit was fixed in the log-logistic model using the mean response of the respective SC. Effect concentrations with 10% (EC_10_) for DT and 50% effect (EC_50_) for ER were derived from the fitted models, and their 95% confidence intervals (CI) were obtained with the implemented function “ED” of the “drc” package using the delta method and the t-distribution. Significant differences between EC_x_ values from different tests were assumed if the respective 95% CI did not overlap.

To assess the influence of test parameters temperature and substrate on ER and DT, SCs were compared between tests in the software Statistica version 13.3 (TIBCO Software 2017) by *t* tests (two-sided, *α* = 0.05).

## Testing of azadirachtin

### Test performance

The formulation ‘Bio-Schädlingsfrei Neem’ (Bayer CropScience) is a concentrated fluid emulsion with a concentration of 10 g azadirachtin/l. Several range-finding tests (RFT) were conducted to determine the range of toxicity of azadirachtin to larvae of *B.* *impatiens* for setting the concentrations in the concentration–response tests (CRT) to determine the NOEC, LOEC and EC_x_ values for ER and DT. RFT and CRT were conducted with oatmeal flakes (shredded at 4 mm) and acetone as the solvent for the reference substance. The solution was applied homogeneously to oatmeal flakes, which were then placed under a fume hood to evaporate the solvent, leaving only the active substance (a.s.) loaded to the oatmeal flakes. Substrate, solution and oatmeal flakes were prepared for each treatment. The first three RFTs were suitable to refine the range of concentrations and help improve the experimental protocols. The concentrations and design of the fourth RFT and the following CRT is given in Table 2. Since C0 and SC did not significantly differ in the RFT for either ER or DT, no C0 was prepared for the CRT. To compare the effect of azadirachtin between CFS and TAS, another CRT with *B. impatiens* in TAS was conducted with the same concentrations as in the previous CRT with CFS, but including an additional higher concentration of 1184 µg a.s./g oatmeal considering the results from the previous CRT.

**Table 2:** Tests conducted with the reference substance azadirachtin

Test	C0	SC	C1	C2	C3	C4	C5	C6	Substrate	Temperature	No. of replicates
RFT	0	0	1	5	25	125	625	/	CFS	25 °C ± 2 °C	4
CRT1	/	0	37	74	148	296	592	/	CFS	20 °C ± 2 °C	4 (8 in SC)
CRT2	/	0	37	74	148	296	592	1184	TAS	20 °C ± 2 °C	4 (8 in SC)

*RFT* range-finding test, *CRT* concentration–response test, *C0* control without solvent, *SC* solvent control, *C1–C6* concentrations [µg a.s./g oatmeal], *CFS* coconut fiber substrate, *TAS* tropical artificial soil

### Test results and discussion

**Fig. 2:**
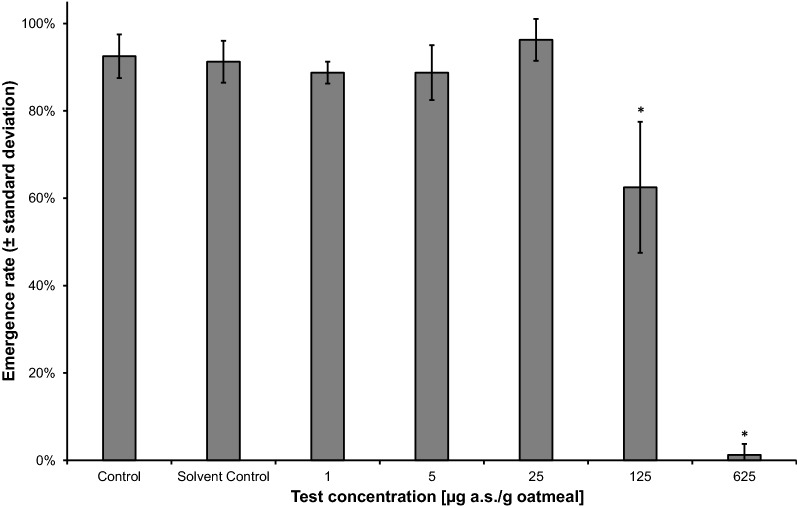
Effect of neem oil (a.s. azadirachtin)-spiked oatmeal in coconut fiber substrate at 25 °C on the emergence rate (± standard deviation) of *Bradysia impatiens*. *Significant difference between treatment and solvent control (Fisher’s exact binomial test with Bonferroni correction, one-sided smaller, *p* ≤ 0.05). *a.s.* active substance

**Fig. 3:**
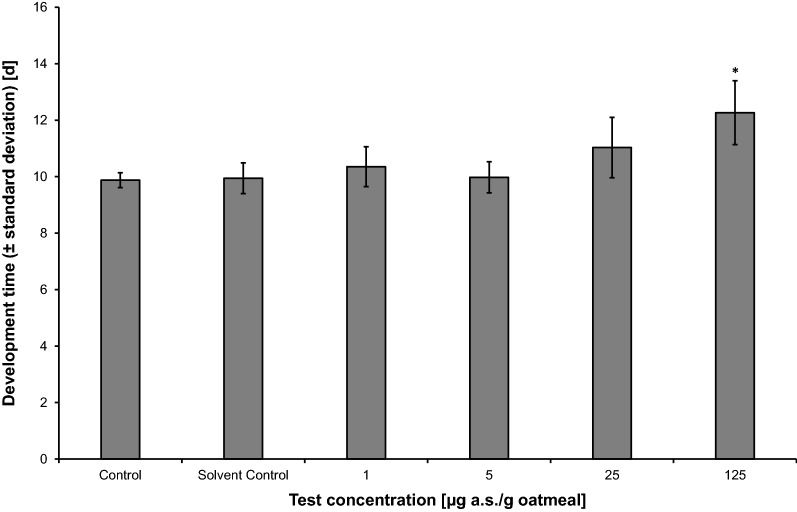
Effect of neem oil (a.s. azadirachtin)-spiked oatmeal in coconut fiber substrate at 25 °C on the development time (± standard deviation) [d] of *Bradysia impatiens*. *Significant difference between treatment and solvent control (Williams *t* test, one-sided greater, *p* ≤ 0.05). *a.s.* active substance

The fourth RFT with a spacing factor of 5 and performed at 25 °C ± 2 °C resulted in a NOEC of 25 µg a.s./g oatmeal and an LOEC of 125 µg a.s./g oatmeal for both ER and DT (Figs. 2, 3). For ER, the coefficient of variation (CV) in the SC was 5.2%; for DT, the CV in the SC was 2.7% and the minimum detectable difference (MDD) to the SC was 10.5–11.2%. However, due to the wide spacing factor, these effect values could not be considered reliable. The EC_50_ for the ER was calculated as 160 µg a.s./g oatmeal (95% CI: 132–188 µg a.s./g oatmeal) and was used to further narrow down the concentration range for the following CRT.

**Fig. 4:**
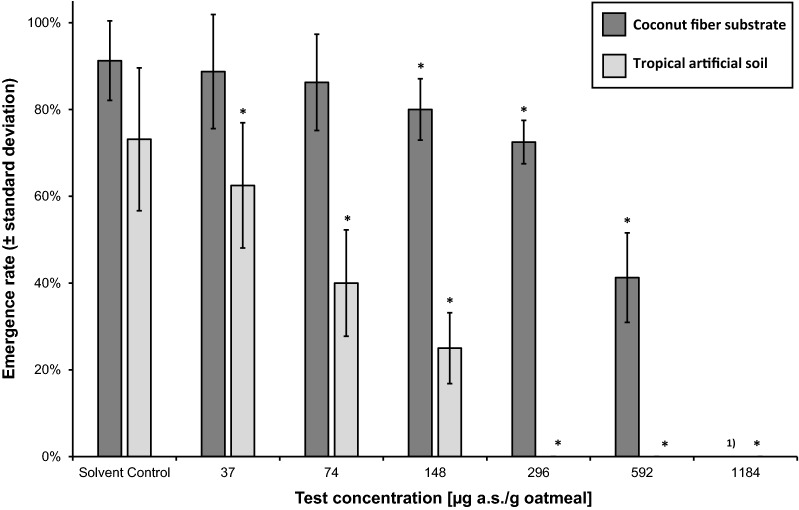
Effect of neem oil (a.s. azadirachtin)-spiked oatmeal in coconut fiber substrate and tropical artificial soil at 20 °C on the emergence rate (± standard deviation) of *Bradysia impatiens*. *Significant difference between treatment and solvent control (*χ*^2^-test with Bonferroni correction, one-sided smaller, *p* ≤ 0.05). *a.s.* active substance. ^1)^Concentration not tested

**Fig. 5:**
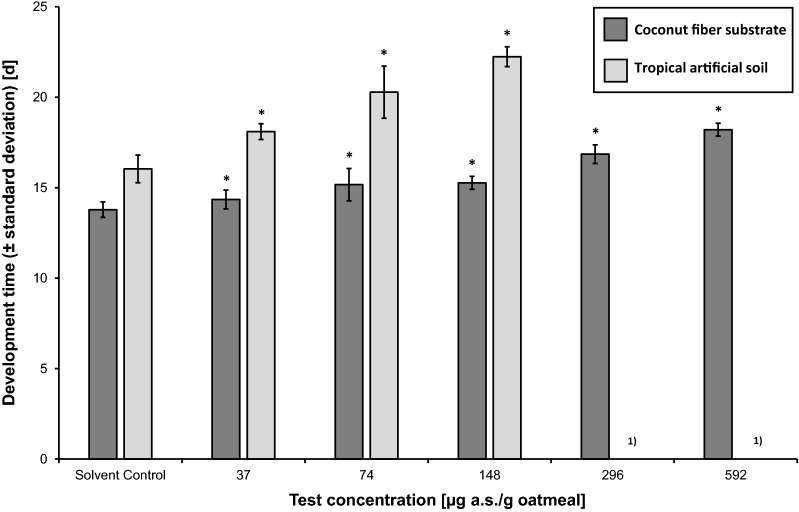
Effect of neem oil (a.s. azadirachtin)-spiked oatmeal in coconut fiber substrate and tropical artificial soil at 20 °C on the development time (± standard deviation) [d] of *Bradysia impatiens*. *Significant difference between treatment and solvent control (Williams *t* test, one-sided greater, *p* ≤ 0.05). *a.s.* active substance. ^1)^No emergence

The CRT performed with CFS at 20 °C ± 2 °C revealed an NOEC of 74 µg a.s./g oatmeal and an LOEC of 148 µg a.s./g oatmeal for the ER (CV = 10.0%; Fig. 4). The EC_50_ for the ER was calculated as 565 µg a.s./g oatmeal (95% CI: 413–717 µg a.s./g oatmeal). The NOEC for DT could not be determined and was considered to be < 37 µg a.s./g oatmeal (CV = 3.1%; MDD = 4.0–4.2% of SC; Fig. 5).

The EC_50_ for the ER in the CRT using TAS was calculated as 88.6 µg a.s./g oatmeal (95% CI: 72.3–105 µg a.s./g oatmeal; CV = 22.5%). An NOEC for both ER and DT could not be determined and was therefore considered to be < 37 µg a.s./g oatmeal and similarly for the LOEC at ≤ 37 µg a.s./g oatmeal (Figs. 4, 5). For DT, the CV in the SC was 4.8% and the MDD to the SC was 5.7–6.1%.

**Fig. 6:**
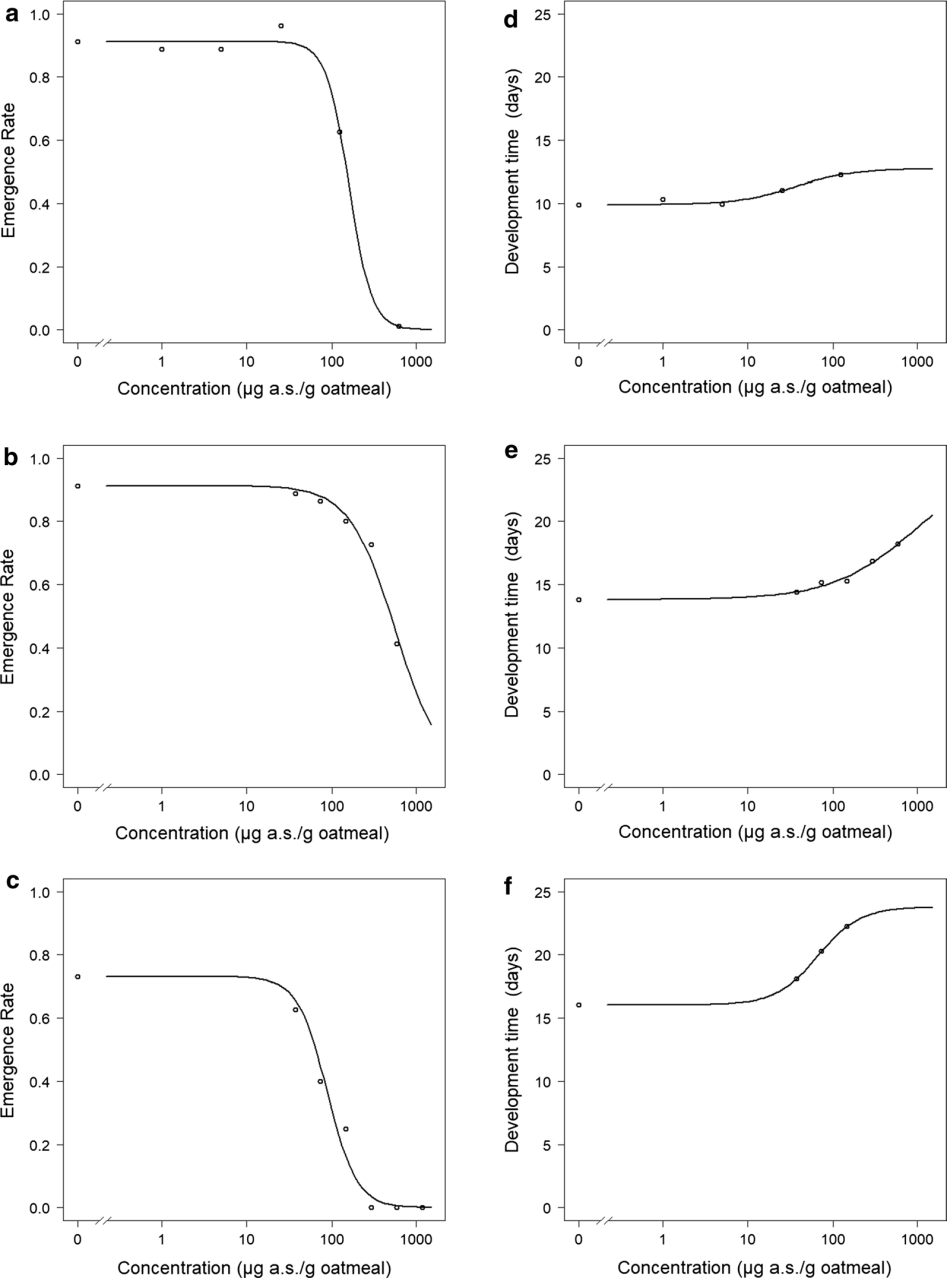
Concentration–response curves for the effect of azadirachtin on *B. impatiens*. **a** Emergence rate (ER) in coconut fiber substrate (CFS) at 25 °C; **b** ER in CFS at 20 °C; **c** ER in tropical artificial soil (TAS) at 20 °C; **d** development time (DT) in CFS at 25 °C; **e** DT in CFS at 20 °C; **f** DT in TAS at 20 °C

The test results are summarized in Table 3, and the concentration–response curves are given in Fig. 6.

**Table 3:** Test results with the reference substance azadirachtin

Test	Emergence rate				Development time				%MDD
	CV in SC [%]	NOEC	LOEC	EC_50_ (95% CI)	CV in SC [%]	NOEC	LOEC	EC_10_ (95% CI)	
RFT	5.2	25	125	160 (132–188)	2.7	25	125	6.19 (0–36.5)	10.5–11.2
CRT1	10.0	74	148	565 (413–717)	3.1	< 37	≤ 37	79.5 (78.8–238)	4.0–4.2
CRT2	22.5	< 37	≤ 37	88.6 (72.3–105)	4.8	< 37	≤ 37	18.7 (7.60–29.9)	5.7–6.1

*RFT* range-finding test, *CRT* concentration–response test, *CV* coefficient of variation, *SC* solvent control, *NOEC* no-observed-effect concentration, *LOEC* lowest-observed-effect concentration, *ECx X* % effective concentration, *CI* confidence interval, *MDD* minimum detectable difference

The comparison between the SCs of RFT, performed at 25 °C, and CRT1 (20 °C) by *t* test revealed that there was no significant difference in ER (91% in both tests; *p* > 0.05), while DT was significantly lower at 25 °C (9.9 days) compared to 20 °C (13.8 days; *p* < 0.0001). This is consistent with the temperature dependency of life cycle parameters described above [48]. The NOEC and LOEC for ER in the final CRT in CFS were comparable with the results from the previous RFT, while the LOEC was clearly lower for DT. As the respective CI did not overlap, both the EC50 for ER and the EC10 for DT were significantly lower at 25 °C compared to 20 °C. The influence of temperature on the interaction of DT of *B. impatiens* and toxicity of azadirachtin cannot currently be explained and would require further testing. A higher toxicity of PPP at higher temperature has also been observed for Collembola, at least as long as the respective test species is adapted to temperate conditions (*Folsomia candida*), while species occurring in Mediterranean or even tropical regions (*Sinella curviseta*) reacted the other way around (e.g., [1, 2]).

Regarding test substrates, ER was significantly lower (73% vs. 91%; *p* = 0.016) and DT significantly higher (16.0 d vs. 13.8 d; *p* < 0.001) in the SCs of the CRT performed with TAS compared to CFS. Again, the CI of the EC50 for ER and the EC10 for DT did not overlap and were thus significantly lower in TAS compared to CFS. This could be due to the fact that DT was already reduced in the TAS controls compared to CFS and, hence, the properties of TAS might have posed an additional stressor leading to a higher sensitivity.

In all three tests, the DT showed a clearly higher sensitivity than the ER; therefore, we consider DT a useful additional test end point. However, it should not be used as the primary end point, as its ecological relevance is less clear than for ER. While for dung-breeding Diptera a delay in DT had a potentially detrimental influence on food availability and thus overall emergence success (e.g., [28], we know of no similar relationship for Sciaridae.

Recommendations for an expected range of NOEC/LOEC or EC_50_ values for the effect of azadirachtin on ER and/or DT of *B. impatiens* at different temperatures can be derived after a full ring test has been performed.

## Conclusions and outlook

A functional basic test design for a new ecotoxicological test method for the ERA of GMP has been successfully developed and the test design is now ready for a full standardization process. In this way, one of the first standardized ecotoxicity tests specifically tailored to GMP and plant-incorporated substances can be realized.

The final test design should be in line with the recommendations of EFSA for testing non-target organisms [12] and must ensure sufficient statistical power to detect the relevant effect level [36]. To do so, the Federal Agency for Nature Conservation commissioned a follow-up R&D project starting in 2018, which will amend the current test design and perform interlaboratory comparison tests followed by a full ring test [35]. Although *B. impatiens* would be a suitable test species, the new project aims to adapt the test procedure to *Lycoriella castanescens* (Diptera: Sciaridae) which is abundant in agricultural fields and therefore more relevant for the risk assessment. In the course of this follow-up project, the method will also be applied for the assessment of actual GMP material. Assuming that the results from the ring test confirm the replicability, sensitivity, practicability, and robustness of the new test method, either an OECD guideline or a guidance document will be prepared.

Recently, the European Food Safety Authority (EFSA) Panel on Plant Protection Products and their Residues (PPR) published an Opinion Paper in which an extended test battery for agricultural land is explicitly required, with a focus on organism groups not covered to date [14]. The use of sciarid flies as test organisms is in line with these recommendations as EFSA stresses the ecological role of dipteran larvae albeit the fact that no standardized methods are yet available. The described test design here is suitable not only for GMP, but also for other stressors such as systemic PPP or veterinary pharmaceuticals that reach the soil by using manure as fertilizer on arable fields. As ecotoxicity tests for those substances are in demand [46], it is planned to include those stressors in the standardization process.

## Abbreviations

a.s.: active substance
*Bt*: *Bacillus thuringiensis*
C0: control without solvent
CFS: coconut fiber substrate
CI: confidence interval
CRT: concentration-response test
CV: coefficient of variation
DR: development rate
DT: development time
EC_10_: 10% effective concentration
EC_50_: 50% effective concentration
EFSA: European Food Safety Authority
ER: emergence rate
ERA: environmental risk assessment
EU: European Union
GMP: genetically modified plant
JKI: Julius-Kühn-Institute
LOEC: lowest-observed-effect concentration
LUFA: Landwirtschaftliche Untersuchungs- und Forschungsanstalt
MDD: minimum detectable difference
NOEC: no-observed-effect concentration
NTO: non-target organism
OECD: Organisation for Economic Co-operation and Development
PDA: potato dextrose agar
RFT: range-finding test
SC: solvent control
TAS: tropical artificial soil
